# Effects of Social Support on Reducing Acculturative Stress-Related to Discrimination between Latin and Asian Immigrants: Results from National Latino and Asian American Study (NLAAS)

**DOI:** 10.9734/JAMMR/2018/42728

**Published:** 2018-08-16

**Authors:** Jung Hye Sung, Jae Eun Lee, Ji-Young Lee

**Affiliations:** 1 School of Public Health, Jackson State University, Mississippi, USA.; 2 College of Science, Engineering and Technology, Jackson State University, Mississippi, USA.; 3 Department of Public Health Sciences, University of Miami Miller School of Medicine, Florida, USA.

**Keywords:** Acculturative stress, family cohesion, discrimination, social network, immigrants

## Abstract

The study aimed to determine both the association between perceived racial discrimination and acculturative stress, and the role of social support serves in the association of discrimination with acculturative stress using data on 3,268 immigrants from the National Latino and Asian Study. Perceived racial discrimination was measured by nine items asking how often the respondent experienced discrimination. Acculturative Stress was defined by nine items designed to measure the stress felt as a result of adapting one’s own culture with a host culture. Buffering effects were determined by the statistical interaction within the multiple linear regression models while controlling for the demographic variables.

While Latin American immigrants were less likely to perceive discrimination than Asian American immigrants did (p=0.0309), they had higher acculturative stress (p=0.0005) and higher levels of social network (p<0.0001). However, there was no significant difference in family cohesion between races/ethnicities. In both groups, acculturative stress was positively related to discrimination and conversely, negatively associated with a social network. Higher levels of social network were significantly related to less acculturative stress in both groups. Our study also found that neither social network nor family cohesion served a role to buffer the effect of the relationship between discrimination and acculturative stress among Asian American immigrants. However, family cohesion alone buffered the relationship between discrimination and acculturative stress among Latin American immigrants (p=0.0184).

Since we found that family cohesion served as a buffering factor in reducing the acculturative stress that is associated with discrimination among Latin immigrants, future social programs designed to enhance social support may reduce acculturative stress among Latin immigrants experiencing high levels of discrimination.

## 1. INTRODUCTION

The U.S. accommodates a variety of diverse cultures and ethnic groups. Immigrants make up a significant component in the United States (U.S.). population, comprising of nearly 44 million individuals, or 13.5% of the total population in 2016 [1]. Accounting for 51% of all immigrants, Latin Americans represent the largest segment of the U.S. immigrant population, with Asian Americans following at 30%.

Despite their substantial demographic compositions across the U.S., racial discrimination against immigrants exists in society. The prevalence of perceived discrimination among Latin Americans has been reported to be 52%, while 65% of young Latin Americans have experienced racial or ethnic discrimination [2]. According to a survey from the National Public Radio, the Robert Wood Johnson Foundation, and Harvard T.H. Chan School of Public Health, about a third of Asian Americans have personally experienced racial or ethnic discrimination (32%) and people making negative assumptions or insensitive or offensive comments about their race or ethnicity (35%) [3].

Previous studies have consistently shown that exposure to racial discrimination is strongly associated with and have implications on both mental health and physical health, including stress, depression, eating disorders, alcohol or substance abuse, and hypertension [4–10].

As with racial discrimination, immigrants continually experience an added layer of psychological stress: acculturative stress. Acculturation has widely been recognized as a process of cultural and psychological changes that occurs when foreign-born individuals are exposed to a new and unfamiliar culture, adopting the behaviors, attitudes, and values of the host culture. Acculturative stress occurs when immigrants experience the stress that arises from the struggle to reconcile the culture of their origin with the host culture [11]. Acculturative stressors can include limited understanding of a new language, the pressures of acquiring a new language, perceived cultural incompatibilities, and cultural self-consciousness [12–14]. Studies on the process of acculturation and corresponding acculturative stress among immigrants have shown to pose serious health and psychosocial consequences, particularly linked with mental health problems [15–18].

Much evidence has supported the association between racial discrimination and acculturative stress. Dawson et al. found that both daily discrimination and major racist events were significantly related to acculturative stress among Dominican women [19]; similarly, Torress et al. found an association between perceived discrimination and acculturative stress in Latin American adults in Midwestern cities [20]. Bekteshi and Hook also found that perceived discrimination impacted acculturative stress in Latin American immigrants [21]. Moreover, a recent study found that Asian immigrant participants reported higher acculturative stress and perceived discrimination compared to all other racial/ethnic groups [22]. The cumulative impact of both racial discrimination and acculturative stress can bear further psychological health implications among immigrants.

Social support is the perception and actuality that one receives supportive resources from other people in their social network, such as emotional, financial, or informational support, or companionship. Leong et al. [23] found that low engagement with social network and low levels of family cohesion increased the risk of anxiety disorders in Asian American immigrants and Singh et al. found that high family social support decreased psychological distress in Asian American immigrants [24–25]. Prior studies have shown the moderating or buffering effect of social support on mental health status, such as depression, stress, anxiety, or feelings of distress [24–28]. However, despite some evidence of an association between racial discrimination and acculturative stress, limited attempts have been made to investigate the racial differences in the buffering effect of social support on reducing acculturative stress related to discrimination. Thus, this study aimed to determine 1) the association between perceived racial discrimination and acculturative stress; 2) the role of social support in the association between discrimination and acculturative stress; and 3) the differences in the buffering effect of social support on reducing acculturative stress associated with discrimination between Asian and Latin American immigrants. Data are drawn from 3,268 immigrants from the National Latino and Asian Study.

## 2. METHODS

### 2.1 Data Source

This study used the data from the National Latino Asian American Study (NLAAS), the first nationally representative community household survey of Asian and Latin Americans conducted between May 2002 and December 2003 [29]. The NLAAS is based on a stratified area probability sample design, and the survey populations for the NLAAS included all Asian and Latin American adults, 18 years of age and older in the U.S. and Washington, D.C. Details on sampling and procedures are described elsewhere [29–30].

Participants born in U.S. or not identified were excluded from this study. After exclusion from the NLAAS, a total of 3,268 immigrants (1,639 Latin-and 1,629 Asian-Americans) were included in the analyses. Latin Americans included ethnic subgroups of Cuban, Puerto Rican, Mexican, and other Latino, whereas Asian Americans included ethnic subgroups of Vietnamese, Filipino, Chinese, and other Asians.

### 2.2 Measures

Perceived discrimination was measured by a “9-item day-to-day experiences” tool asking how often the respondent experienced discrimination in the past year. Nine items for perceived discrimination are as follows: treated with less courtesy, treated with less respect, received poorer service at restaurants or stores, treated as not smart, treated in a way that people acted afraid of you, treated as dishonest, treated in a way that people acted better than you, were called names or insulted, and were threatened or harassed. Each item was measured on a 6-point Likert-type scale ranging from 1 (almost every day) to 6 (never). Each item score was coded reversely in order, such that higher values represented higher levels of perceived discrimination. An average of nine items was calculated and used in this study for the perceived discrimination scale score, which had a Cronbach’s alpha (α) of 0.91.

Acculturative Stress was defined by the sum of nine items designed to measure the stress felt as a result of adapting one’s own culture with a host culture. Each item had dichotomized responses (yes = 1 or no = 0). All items were summed, with higher values representing higher acculturative stress (α=0.67). Nine items for acculturative stress are as follows: felt guilty about leaving family/friends in country of origin, avoided health service due to fear of immigration officials, felt same respect in U.S. as in country of origin (reverse coded), had limited contact with family and friends, had difficult interactions due to difficulty with English language, was treated badly due to poor or accented English, had difficulty finding work due to Latino or Asian descent, was questioned about legal status, and perceived deportation by going to a social or government agency.

Family cohesion was measured by the extent of agreement for each of the ten statements describing emotional support, belongings, loyalty, respect etc. (α =0.93). Ten items for family cohesion are as follows: family members respect one another, family shares values, things work well as family, family trusts and confides in each other, family feels loyal to family, family is proud of family, family expresses feelings with family, family likes to spend free time with each other, family feels close to each other, and family togetherness is important. Each item was measured on a 4-point scale ranging from 1 (strongly agree) to 4 (strongly disagree). Each item score was coded reversely in order such that higher values represented higher levels of family cohesion. An average of the 10 items was calculated and used for the family cohesion scale score.

Social network was assessed by averaging across eight items including social network among family and friends. Four items for social network were measured on a 4-point Likert-type scale ranging from 1 (strongly agree) to 4 (strongly disagree) and the other four items were measured on a 5-point Likert-type scale ranging from 1 (always) to 5 (never). Each item score was coded reversely in order such that higher values indicated greater social network with family and friends (α =0.75). Eight items assessed on social network are as follows: frequency of talking on phone with family not living with you, extent of relying on relatives for serious problems, extent of relying on relatives for discussing worries, frequency of talking on phone with friends, extent of relying on friends for serious problem, extent of opening up to friends to talk about worries, frequency of informing families about problems/worries, frequency of informing someone else about problems/worries.

Socio-demographic variables age, gender and marital status as well as socio-economic variables employment status and education were also used in the analyses.

### 2.3 Analysis Plan

Descriptive statistics of the demographic characteristics were measured by using a combination of a weighting factor in the NLASS and PROC SURVEYFREQ in SAS. Rao–Scott χ2 tests, which were adjusted for the complex sampling design by using PROC SURVERYFREQ, were conducted to assess any differences by races/ethics in the demographic characteristics. Multiple linear regression analyses were conducted to estimate the differences in major indices between races/ethics after controlling for age, gender, employment status, marital status, and education.

To assess the association between acculturative stress, discrimination, and social support by races/ethnicities, multiple linear regression analyses were also conducted with acculturative stress as the dependent variable after controlling for other covariates in Model 1. Buffering effects were determined by the statistical interaction within the multiple linear regression models while controlling for the demographic variables. To assess the buffering effects on the relationship between discrimination and acculturative stress by races/ethnicities, Model 2 and Model 3 included the interaction of discrimination and social network and the interaction of discrimination and family cohesion, respectively, in addition to all variables in Model 1. Standard errors of the multiple linear regression analyses were estimated using Taylor series linearization (TSL), implemented with the SAS SURVEYREG, to incorporate complex sampling design and post-standardization. All analyses were conducted using SAS version 9.4 (SAS Institute, Inc).

## 3. RESULTS

Table 1 describes the descriptive statistics for the demographic characteristics of Asian and Latin American immigrants in this study population. While Latin American immigrants were more likely to be male (p=0.032) and younger ((p<.0001) than their Asian American counterparts, Asian American immigrants were more likely to be highly educated (p<.0001) than their Latin American counterparts. Marital status and employment status were not different between races/ethnicities. Although frequency was not weighted, percentage and Rao–Scott χ2 tests accounted for sampling design and weights.

Table 2 presents the mean of major indices stratified by races/ethnicities and the results of statistical tests using multiple linear regression analyses to estimate the differences in major indices between Asian and Latin American immigrants after controlling for age, gender, employment status, marital status, and education. Latino immigrants had higher acculturative stress (p=0.0005) and higher social network (p<0.0001) than did Asian American immigrants. However, Latin American immigrants had lower perceived discrimination than did Asian American immigrants (p=0.0309). No difference in family cohesion (p=0.3510) existed between Asian and Latin immigrants.

Table 3 presents that acculturative stress was related to discrimination, social support, and family cohesion in both Asian and Latin American immigrants. There was significant difference in acculturative stress between the two races/ethnicities (Model 1, p<0.0001). Latino immigrants had higher acculturative stress compared to Asian American immigrants after controlling for main indices and demographic variables. There was a strong positive association between discrimination and acculturative stress in both races/ethnicities in Model 1(p<0.0001). While acculturative stress was negatively associated with social network in both groups, acculturative stress was not significantly related to family cohesion after controlling for other variables in both groups. The buffering effects of social network and family cohesion on the relationship between discrimination and acculturative stress, stratified by races/ethnicities are shown in Model 2 and Model 3. Among Asian American Immigrants, neither social network (p=0.8178) nor family cohesion (p=0.2262) buffered the association of discrimination and acculturative stress. On the contrary, family cohesion buffered the relationship between discrimination and acculturative stress among Latin American immigrants (p=0.0184). However, social network did not buffer the effect of discrimination and acculturative stress among Latin American immigrants (p=0.3257).

## 4. DISCUSSION

To our knowledge, our study is the first to determine differences in the buffering effects of social support on reducing acculturative stress related to discrimination between Asian and Latin American immigrants. Overall, our study found that while Latin American immigrants were less likely to perceive discrimination than Asian American immigrants did, they had higher acculturative stress and higher levels of social network. However, there was no significant difference in family cohesion between races/ethnicities. In both Asian and Latin American immigrant groups, acculturative stress was positively related to discrimination and conversely, negatively associated social network. Higher levels of network were significantly related to less acculturative stress in both groups. Our study also found that neither social network nor family cohesion served a role to buffer the effect of the relationship between discrimination and acculturative stress among Asian American immigrants. However, family cohesion alone buffered the relationship between discrimination and acculturative stress among Latin American immigrants.

Our study had consistent findings with recent prior studies on the association between acculturative stress and perceived discrimination, family cohesion, and social network [21,23,24]. Bekteshi and Hook found that perceived discrimination impacted acculturative stress in Latin American immigrants, and also showed that duration of living in the U.S. and perceived discrimination interacted in predicting acculturative stress in Latin American immigrants [21]. Leong et al. found that low engagement with social network and low levels of family cohesion increased the risk of anxiety disorders in Asian American immigrants, and that high levels of family cohesion were a significant protective factor against depressive disorders in Latin American immigrants [23]. Finally, Singh et al. found that high family social support decreased psychological distress, and that friend social support buffered the relationship between discrimination and psychological distress in Asian American immigrants [24]. However, none of these studies assessed the buffering effects of family cohesion and social network on acculturative stress and discrimination in Latin and/or Asian American immigrants.

The results of the present study contradict the results of one previous study assessing differences in acculturative stress and perceived discrimination between Asian and Latin American immigrants [22]. Gomez et al. reported that among 969 university students (ages 18–25), Asian participants had higher acculturative stress and perceived discrimination compared to all other racial/ethnic groups among 969 university students [22]. Accounting for this, we performed supplementary analysis among 269 immigrants with at least a high school education between the ages of 18 and 25 years in our study and found that Asian American immigrants had less acculturative stress compared to Latin American immigrants and no difference existed in perceived decimation between the two groups. One possible reason for the contrasting results between ours and Gomez et al.’s study may be related to the different birthplaces between the U.S. and other countries. While we included only participants who were born in another country outside of the U.S., Gomez’s study included participants born both within and outside the U.S. This suggests that first generation immigrants (such as those included in our study) may have already established an identity with their native culture and do not perceive acculturation as a difficult divide to overcome, whereas those who were born within the U.S. (such as those included in Gomez et al,’s study) felt increasing tensions in cultural identity throughout their life course. Another possible reason may be due to the differences in education levels. Gomez’s study included only public university students in the northeastern part of the U.S. Although our supplemental analysis was limited to those with at least a high school education, 35% consisted of high school graduates and 25% were college graduates. The Asian American immigrants in Gomez et al.’s study were actively in college, which in itself is a formative period when students are searching and developing their self-concepts [31]. Thus, the Asian American immigrant college students in their study sample may have experienced further tension in their identities during that developmental time.

We also found evidence of differences in the buffering effect of social network and family cohesion on the relationship between perceived discrimination and acculturative stress between Asian and Latin American immigrants. While family cohesion buffered the relationship between perceived discrimination and acculturative stress among Latin American immigrants, it did not play the same buffering role for Asian American immigrants. In contrary to the hypothesis associated with the buffering effect of social network on the association of perceived discrimination and acculturative stress, our study found that it did not buffer the association of perceived discrimination and acculturative stress in either Latin or Asian American immigrants. Even upon conducting supplementary analyses to examine the buffering effect of social network and family cohesion among the four Asian American immigrant sub-groups (Vietnamese Filipinos, Chinese, and others), no buffering effect was present among any and all four Asian American immigrant sub-groups. One such possibility may be due to Asian American’s adherence to Asian cultural values. Kim and colleagues suggested that although significant within-group differences among Asian Americans exist, they all share significant common cultural values and beliefs (e.g., collectivism, conformity to norms, deference to authority, emotional self-control, family recognition to achievement, etc.) [32]. Kim and Omizo further noted that adhering to Asian cultural values may play a role in developing a positive self-concept for Asian American immigrants [33]. Thus, it may be that Asian American immigrants in our sample had high adherence to Asian cultural values and have embedded strong social network and family cohesion that already exists within Asian values, serving no significant buffering effect. Further research is warranted to identify the reasons why neither social network nor family cohesion buffered the effect of discrimination and acculturative stress among Asian American immigrants.

Our study has a couple of limitations related to the data. First, the measures of study variables were based on self-reports and may have potentially biased the relations among significant indices and some constructs. Second, because NLAAS was a cross-sectional survey, causal inferences were unable to be assessed. Lastly, because years elapsed since the survey for NLAAS was conducted, the perceived discrimination or acculturative stress based on races/ethnicities might have changed.

## 5. CONCLUSION

Despite these limitations, our study is the first to determine differences in the buffering effects of social support on reducing acculturative stress related to discrimination between Asian and Latin American immigrants. Our findings suggest that family cohesion was found to have a buffering effect on reducing acculturative stress associated with discrimination among Latin American immigrants. One implication of these findings is that social programs enhancing social support may reduce acculturative stress among Latin American immigrants experiencing high discrimination. Future programs should assess individuals’ support systems and attempt to improve the quality of their existing social support relationships to help ameliorate the psychological changes that occur when individuals are exposed to another culture. Further, cultural tailoring of future programs is warranted, as different support systems (i.e., social network and family cohesion) function differently between Asian and Latin American immigrants.

## Figures and Tables

**Table 1.** Demographic characteristics of study population

	All (n=3268)		Asian	(n=1,639)	Latino	(n=1,629)	p-value^*^
	N	%	N	%	N	%	
**Age**							
Age 18–30	766	30.0	372	24.5	394	32.5	<0.0001
Age 31–40	845	27.7	425	25.2	420	28.9	
Age 41–50	725	20.0	406	22.8	309	18.7	
Age 51–60	483	11.2	238	13.6	245	10.0	
Age 60+	459	11.1	198	13.7	261	9.9	
**Gender**							
Female	1774	50.1	868	53.5	906	48.5	0.0382
Male	1494	49.9	771	46.5	723	51.5	
**Education status**							
Years of education 0–11	1045	43.5	300	18.7	745	55.2	<0.0001
Years of education 12	6636	18.7	274	16.2	362	19.9	
Years of education 13–15	666	17.9	369	22.0	297	16.0	
Years of education 16+	921	19.9	696	43.1	225	8.9	
**Employment status**							
Not employed	1225	36.4	562	36.6	663	36.3	0.9104
Employed	2043	63.6	1077	63.4	966	63.7	
**Marital status**							
Not married/single	944	28.5	401	25.7	543	29.8	0.0634
Married/cohabitated	2324	71.5	1238	74.3	1086	70.2	

* p-value from Rao–Scott χ2

**Table 2.** Differences in major indices between Asian and Latin American immigrants

Variable	Asian	Latino	Multiple linear regression analyses				
	Mean(95% CL)	Mean(95% CL)	β	S.E	95% CL for β	R^2^	p-value^*^
Acculturative Stress	1.76 (1.68, 1.84)	2.20 (2.11, 2.30)	−0.39	0.11	(−0.60, −0.17)	0.071	0.0005
Discrimination	0.69 (0.65, 0.72)	0.57 (0.53, 0.60)	0.09	0.04	(0.01, 0.18)	0.067	0.0309
Social Network	2.37 (2.34,2.41)	2.61 (2.57, 2.64)	0.03	0.03	(−0.03, 0.08)	0.036	<0001
Family Cohesion	3.73 (3.70, 3.75)	3.69 (3.67,3.72)	−0.24	0.04	(−0.32, −0.16)	0.065	0.351

* p-value from multiple linear regression analysis after controlling for age, gender, employment status, marital status, and education.

**Table 3.** Main and buffering effects on acculturative stress

	All Samples^§^				Asiain				Latino			
	β	95% CL for β	R^2^	p-value^*^	β	95% CL for β	R^2^	p-value^*^	β	95% CL for β	R^2^	p-value^*^
**Model 1**												
Discrimination	0.58	(0.40, 0.75)	0.131	<.0001	0.51	(0.29, 0.74)	0.074	<.0001	0.60	(0.37, 0.82)	0.128	<.0001
Social Network	−0.25	(−0.41,−0.10)		0.0020	−0.29	(−0.46,−0.11)		0.0020	−0.22	(−0.44,−0.01)		0.0409
Family Cohesion	−0.14	(−0.46,0.18)		0.3778	−0.10	(−0.31,0.11)		0.3437	−0.14	(−0.60,0.31)		0.5363
**Model 2**												
Discrimination × Social Network	0.10	(−0.14,0.33)	0.122	0.4519	−0.03	(−0.29, 0.23)	0.074	0.8178	0.14	(−0.14,0.43)	0.129	0.3257
**Model 3**												
Discrimination × Family Cohesion	0.49	(0.14, 0.85)	0.131	0.0046	0.19	(−0.14,0.52)	0.065	0.2262	0.60	(0.13, 1.06)	0.137	0.0184

* p-value from multiple linear regression analysis after controlling for age, gender, employment status, marital status, and education.

§ “Races/Ethnics” variable was included in the models for All samples only along with other controlling variables
